# Establishment and validation of a prognostic signature for pancreatic ductal adenocarcinoma based on lactate metabolism-related genes

**DOI:** 10.3389/fmolb.2023.1143073

**Published:** 2023-06-09

**Authors:** Xin Huang, Chongyu Zhao, Yuanxia Han, Shengping Li

**Affiliations:** ^1^ State Key Laboratory of Oncology in South China, Collaborative Innovation Center for Cancer Medicine, Sun Yat-sen University Cancer Center, Guangzhou, China; ^2^ Department of Experimental Research, Sun Yat-sen University Cancer Center, Guangzhou, China; ^3^ Department of Pancreatobiliary Surgery, Sun Yat-sen University Cancer Center, Guangzhou, China

**Keywords:** pancreatic ductal adenocarcinoma, lactate metabolism, signature, chemotherapy, immune infiltration

## Abstract

**Background:** Pancreatic ductal adenocarcinoma (PDAC) is one of the most aggressive and lethal malignancy with poor prognosis. To improve patient outcomes, it is necessary to gain a better understanding of the oncogenesis and progression of this disease. Metabolic reprogramming, particularly the regulation of lactate metabolism, is known to have a significant impact on tumor microenvironment and could provide valuable insights for the management of PDAC patients. In this study, we aimed to investigate the prognostic potential of lactate metabolism-related genes (LMRGs).

**Methods:** Transcriptomic data of patients with PDAC along with the clinical outcomes were retrieved from The Cancer Genome Atlas database, and the expression data in normal pancreas from Genotype-Tissue Expression dataset were adopted as the normal control. By using Cox and LASSO regression models, we identified key genes that are differentially expressed in cancerous tissues and related to prognosis. To determine the prognostic value of LMRGs in PDAC, we evaluated their clinical significance and model performance using both the area under the receiver operator characteristic curve (AUC) and calibration curves. In addition, we evaluated the drug sensitivity prediction and immune infiltration by using oncoPredict algorithm, single sample gene set enrichment analysis and Tumor Immune Estimation Resource.

**Results:** A total of 123 LMRGs were identified through differential gene screening analysis, among which 7 LMRGs were identified to comprise a LMRGs signature that independently predict overall survival of these PDAC patient. The AUC values for the LMRGs signature were 0.786, 0.820, 0.837, and 0.816 for predicting 1-, 2-, 3- and 5-year overall survival respectively. Furthermore, this prognostic signature was used to stratify patients into high-risk and low-risk groups, with the former having worse clinical outcomes. This observation was further validated through analysis of the International Cancer Genome Consortium database. In addition, lower sensitivity to gemcitabine and infiltration of immune effector cells were observed in the cancer tissue of patients in the high-risk group.

**Conclusion:** In conclusion, our data suggests that a genomic signature comprised of these LMRGs may be a novel predictor of overall clinical outcomes and present therapeutic potential for PDAC patients.

## 1 Introduction

Pancreatic ductal adenocarcinoma (PDAC) is currently one of the most aggressive and lethal malignancy in the world (Siegel et al., 2020). Despite significant advances that have been made in the development of therapeutic strategies such as radiotherapy and pharmacological agents, the 5-year survival rate remains lower than 10%. Notably, PDAC is currently the fourth and sixth leading cause of cancer related death in the US and China, respectively, and it is projected to become the second leading cause of cancer death by 2030 (Rahib et al., 2014). One of the biggest challenges posed in managing patients with PDAC is that majority of them are not diagnosed until late stages due to the lack of symptoms, as well as early screening strategies. Once diagnosed in later stages, a combination of different types of cytotoxic chemotherapies is the first-line treatment strategy for PDAC, alongside radiotherapies or immunotherapies; however, such treatment strategies only afford modest success in improving survival rates (Di Marco et al., 2010; Hidalgo, 2010; Wu et al., 2019).

It has been reported that genetic signatures of mutations on KRAS, TP53, SMAD4 and CDKN2A potentially drive the oncogenic force in the early stages of PDAC progression (Cicenas et al., 2017); however, such genetic signatures have little therapeutic significance except for KRAS, since advances have been made in treating PDAC patients harboring KRASG12D or KRASG12C mutations (Hong et al., 2020; Leidner et al., 2022). Indeed, there is a lack of biomarkers or tools to evaluate the prognosis of PDAC patients, with carbohydrate antigen 19–9 (CA19-9) being the only FDA-approved biomarker used clinically. Taken together, for better management of PDAC patients, there is an urgent need for novel cellular tumor-related genetic signatures to evaluate the progression of the tumor, develop better treatment strategies, and predict the overall clinical outcomes.

Metabolic reprogramming is one of the emerging hallmarks of all cancers (Hanahan and Weinberg, 2000; Hanahan and Weinberg, 2011). Oncogenic mutations reprogram cancer cells to depend on aerobic glycolysis to maintain malignant proliferation and growth. This enables them to metabolize glucose for energy while producing lactate even when oxygen is adequately supplied, a phenomenon known as the Warburg effect (Bose et al., 2021). Such a metabolic feature of cancer cells often leads to the pathological accumulation of lactate, which promotes proliferation and growth of cancer cells (Brown and Ganapathy, 2020; Watson et al., 2021), potentially through translocation into nucleus, regulating key oncogenic activation through epigenetic histone lactylation, or through cell surface GPR81 receptor (Brown and Ganapathy, 2020). Additionally, acidification of the tumor microenvironment inhibits infiltration of immune killer cells and promotes immunosuppressive phenotypes of macrophages, neutrophils or T cells, thus affecting the efficacy of immunotherapy (Deng et al., 2021; Cappellesso et al., 2022).

Importantly, given the significant presence and vital role of lactate in almost all forms of tumors, it has been reported that genetic signature comprised of changes in a host of lactate metabolism-related genes (LMRGs) predicts the immuno-status of tumor microenvironment as well as overall prognosis in hepatocellular carcinoma (Li et al., 2021) and breast cancer (Yang et al., 2022). In the present study, we aim to identify a novel LMRGs signature to predict overall clinical outcomes and provide therapeutic choices for PDAC patients.

## 2 Materials and methods

### 2.1 Transcriptomic data acquisition

RNA transcriptomic sequencing data and corresponding clinical profiles of a total of 185 PDAC patients were retrieved from The Cancer Genome Atlas (TCGA) database (https://portal.gdc.cancer.gov). In addition, due to the limited number of normal pancreas (*n* = 4) in TCGA database, the gene expressions in normal pancreas (*n* = 167) were downloaded as normal control from the Genotype-Tissue Expression (GTEx) as previously described (Huang et al., 2020). Furthermore, we downloaded related expression and survival data of 96 PDAC patients from International Cancer Genome Consortium (ICGC) database (https://dcc.icgc.org/), including PACA-AU cohort (*n* = 77) and PACA-CA cohort (*n* = 19).

### 2.2 Identification of differentially expressed LMRGs

All 294 LMRGs were retrieved from the Molecular Signature Database for this analysis (Supplementary Table S1). To identify specific LMRGs involved in the oncogenesis and progression of PDAC, after exclusion of pathologically non-PDAC data in TCGA, we performed a differential expression analysis in 167 normal tissues (from GTEx) and 163 cancer tissues (from TCGA), which were normalized in R using normalizeBetweenArrays (Cao et al., 2019). Genes with log2 (fold change) > 1 and false discovery rate (FDR) < 0.05 were considered as differentially expressed gene.

### 2.3 Evaluation of potential prognostic values of differentially expressed LMRGs

Patients who had a follow-up time no more than 30 days or had no clinical overall survival (OS) data were excluded, then Cox regression analysis was performed to identify the LMRGs that have potential prognostic value. Additionally, LASSO Cox regression (iteration = 100) model was utilized with the “glmnet” package to prevent overfitting, a low mean-squared error were obtained via lasso.min (Zhang W. et al., 2022a). Finally, the selected LMRGs were subjected to multivariate Cox regression analysis to determine the subset of genes that comprised the LMRGs signature.

### 2.4 Protein expression of LMRGs in PDAC

Protein expression of identified LMRGs in normal pancreas or PDAC were analyzed from the Human Protein Atlas (HPA) (Uhlén et al., 2015), which aims to create a human proteome-wide map through integrated omics technologies.

### 2.5 Detection and evaluation of low-risk vs. high-risk based on LMRGs signature

Riskscore for the current TCGA-PDAC patient cohort were calculated with the following equation: riskscore = expressional levels of LMRG1 * coefficient factor of LMRG1 + expressional levels of LMRG2 * coefficient factor of LMRG2 + … + expressional levels of LMRGn * coefficient factor of LMRGn.

Patients with a calculated riskscore lower than the median value of all patients were designated as “low-risk,” while those with a calculated riskscore higher than the median value were considered to be in the “high-risk” category. PCA and t-SNE analyses were performed to ensure the accuracy of subgroup classification. Subsequently, Kaplan-Meier survival analysis was conducted to compare the OS time between the low-risk and high-risk subgroups.

### 2.6 Evaluation of potential prognostic values of riskscore and other clinical characteristics

Univariate and multivariate Cox regression, as well as receiver operating characteristic (ROC) analysis, were performed to evaluate the potential prognostic values of riskscore and other clinical characteristics, including age, gender, grade, stage, tumor stage (T) and node stage (N). Metastasis status (M) data were excluded due to the high rate of missing values in the TCGA-PDAC dataset. ROC curves were generated using the “survivalROC” package in R.

### 2.7 Establishment of nomograms of prognostic

Nomograms were constructed based on the LMRGs signature and key clinical parameters to predict the 1-, 2- and 3-year survival probabilities for patients in the TCGA-PDAC cohort. Calibration curves were employed to evaluate the consistency between predicted survival rates and actual survival rates.

### 2.8 GO and KEGG enrichment analysis of differentially expressed genes between high-risk and low-risk groups

First, as mentioned above, genes with log2 (fold change) > 1 and false discovery rate (FDR) < 0.05 were considered as differentially expressed gene between high-risk and low-risk group. Second, GO and KEGG pathway enrichment analysis were performed using “clusterprofiler” package in R (version 4) to delineate the cellular, molecular and metabolic pathways that are potentially influenced by the afore-identified LMRGs.

### 2.9 Prediction of the sensitivity of chemotherapies

We evaluated the drug sensitivity against gemcitabine, 5-fluorouracil, oxaliplatin and cisplatin by utilizing the oncoPredict R package as previously reported (Zhang J. et al., 2022b), difference in drug sensitivity between high-risk and low-risk groups was compared by sensitivity score, which was positively correlated with the IC_50_ value of chemotherapy agents (Maeser et al., 2021).

### 2.10 Comparison of immune cell infiltration between low-risk and high-risk groups in PDAC

First, we used the Tumor Immune Estimation Resource (TIMER; http://timer.cistrome.org/) to analyze the correlation between LMRGs expression and immune cell infiltration (including B cell, CD8^+^ T cell, CD4^+^ T cell, macrophage, neutrophil and dendritic cell) in PDAC (Li et al., 2017). Second, we adopted single sample gene set enrichment analysis (ssGSEA) to calculate the level of tumor immune infiltration in the R package GSVA (Hänzelmann et al., 2013). Additionally, ESTIMATE algorithm was employed to infer immune and stromal components for PDAC samples (Yoshihara et al., 2013).

### 2.11 Validation of prognostic value of LMRGs in ICGC

The prognostic value of LMRGs were validated by using previously described ICGC data. First, the riskscore was calculated based on the formula provided by the TCGA cohort. Next, the samples were categorized into high-risk or low-risk groups based on the median riskscore of the ICGC cohort. Time-dependent ROC and KM survival curves were constructed to verify the predictive values of the signature.

### 2.12 Statistical analysis

All analyses were performed using R software (version 4). All sample data were tested for normal distribution; if the dataset was not normally distributed, difference between groups were compared using Wilcoxon rank-sum test or Kruskal–Wallis test. Cox regression model was used to perform univariate or multivariate analysis. The log-rank test was utilized for the evaluation of survival. Significance was defined as a *p*-value <0.05.

## 3 Results

### 3.1 Identification of specific LMRGs in PDAC

**FIGURE 1:**
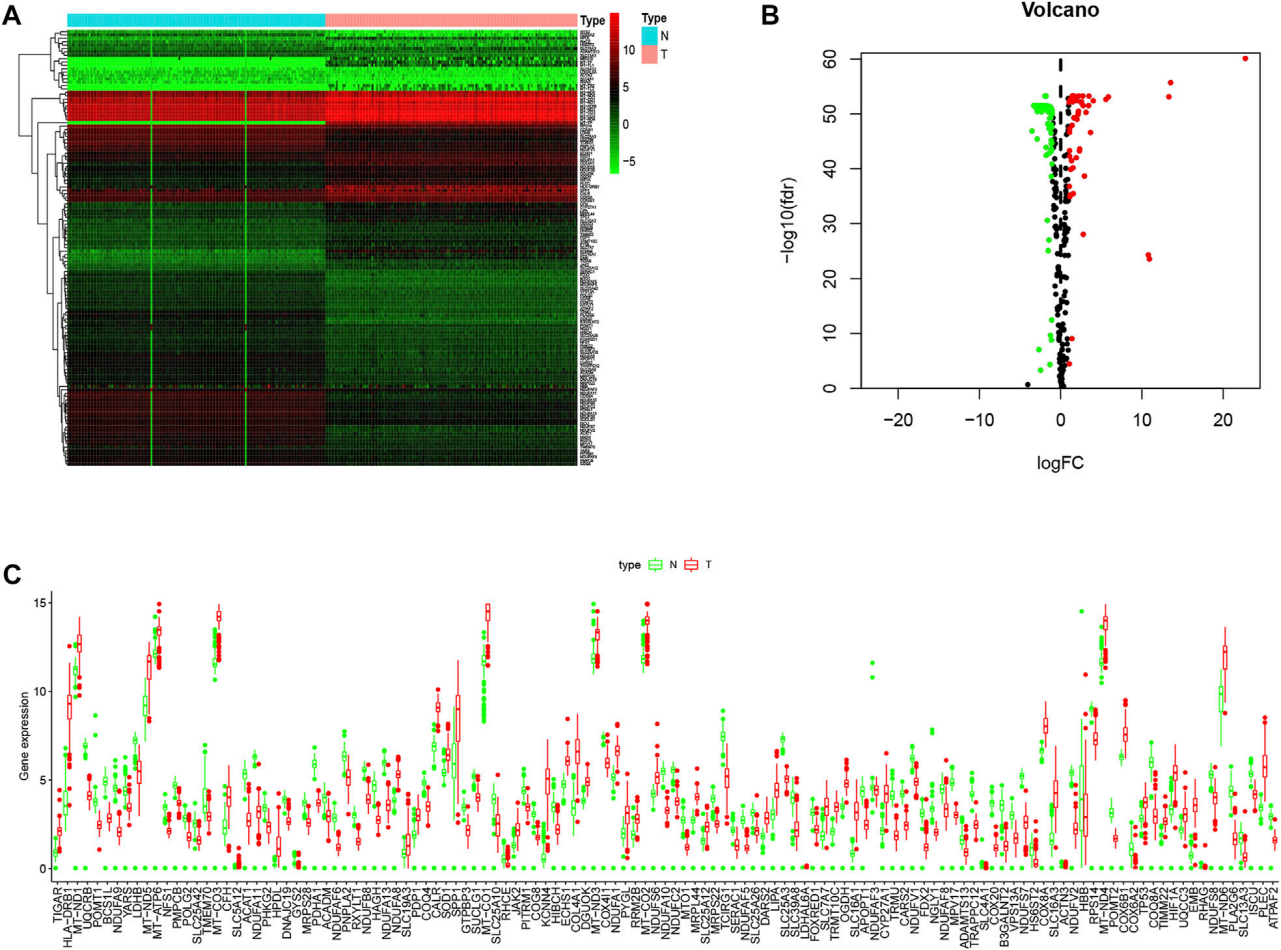
Expression levels of LMRGs in pancreatic tissues. **(A)** Heatmap showing the expression levels of identified LMRGs in pancreatic cancer tissues and normal pancreas. **(B)** Volcano showing the relative expression of identified LMRGs in pancreatic cancer tissues and normal pancreas. **(C)** Boxplot showing the expression level of identified LMRGs in pancreatic cancer tissues and normal pancreas; the *y*-axis shows absolute transcript expression levels measured by FPKM. LMRGs, lactate metabolism-related genes; FPKM, fragments per kilobase of exon per million fragments mapped.

After exclusion of RNA genes, 123 out of the total 294 documented LMRGs were identified through screening for differentially expressed genes using cancer data from TCGA and normal data from GTEx, among which 76 were downregulated while 47 were upregulated. To visualize the relative expressional levels of these LMRGs in normal pancreas and pancreatic cancerous tissues, we generated a heatmap and volcano plot (Figures 1A, B). In addition, we presented the expression level of the identified LMRGs in Figure 1C.

### 3.2 Construction of a prognostic LMRGs-associated model in PDAC

**FIGURE 2:**
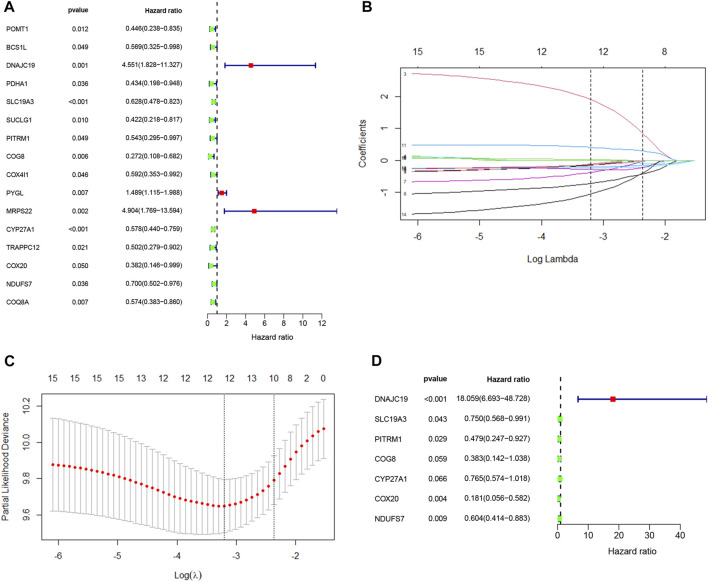
Cox regression analysis and LASSO analysis of LMRGs in PDAC. **(A)** Univariate Cox regression analysis of the identified LMRGs screened 18 prognostic LMRGs in PDAC. **(B,C)** Tuning parameter (λ) selection in LASSO model using cross-validation in PDAC. **(B)** The changing trajectory of each independent variable. The horizontal axis represents the log value of the independent variable lambda, and the vertical axis represents the coefficient of the independent variable. **(C)** Confidence intervals for each lambda. The horizontal axis represents the log value of the independent variable lambda, and the vertical axis represents the error of cross-verification. **(D)** Multivariate Cox regression analysis of LMRGs in PDAC. LMRGs, lactate metabolism-related genes; PDAC, pancreatic ductal adenocarcinoma.

**FIGURE 3:**
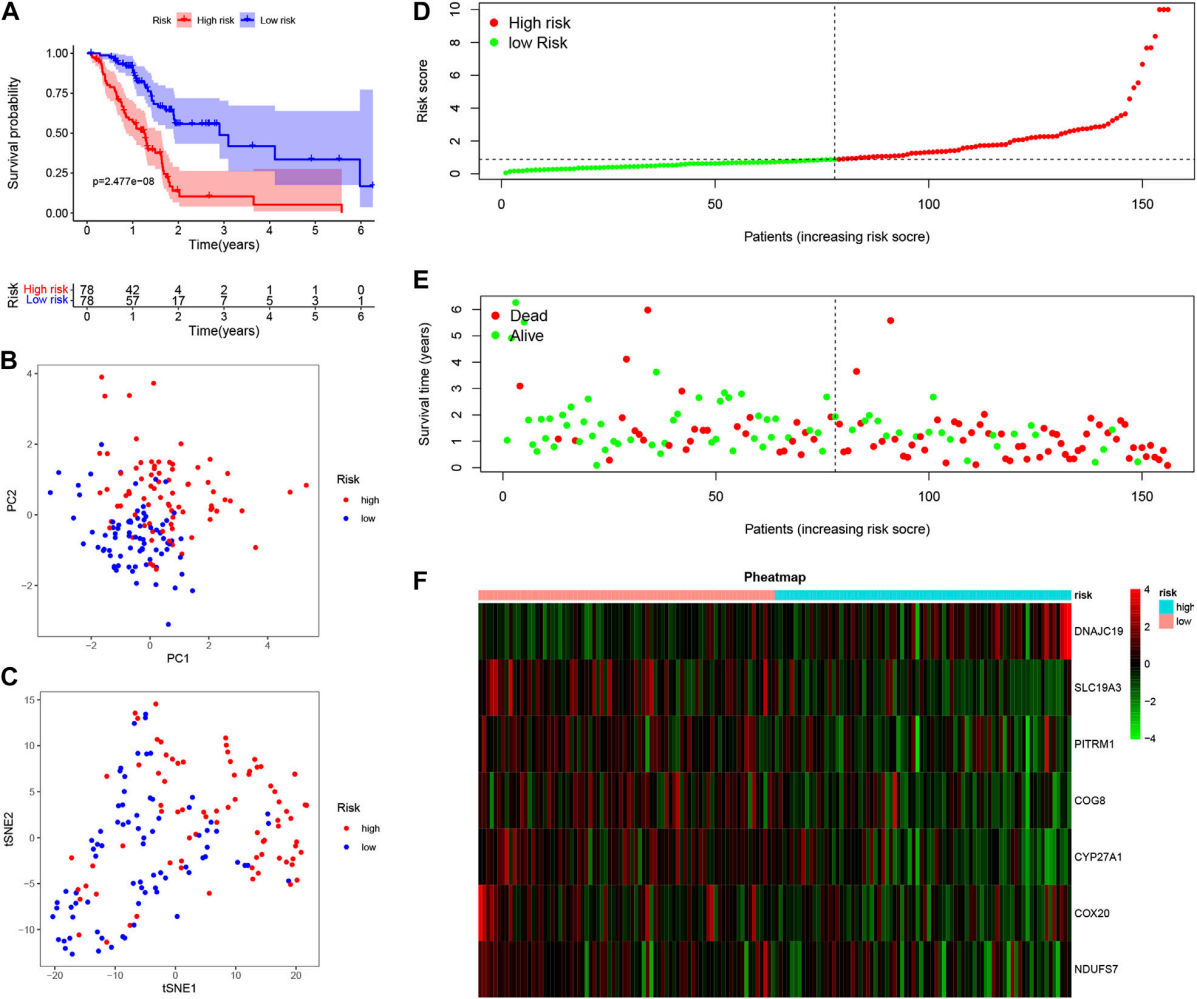
Prognostic value of LMRGs signature in PDAC. **(A)** Kaplan-Meier survival analysis between high-risk and low-risk subgroups based on LMRGs expression in PDAC from TCGA database. **(B,C)** PCA and t-SNE were used to assess whether the samples could be accurately grouped based on the LMRGs scores. **(D)** The riskscore of each pancreatic cancer patient. **(E)** The survival status and survival time of patients in different groups. **(F)** Heatmap of the relative expression of 7 LMRGs in high-risk and low-risk subgroups. LMRGs, lactate metabolism-related genes; PDAC, pancreatic ductal adenocarcinoma; TCGA, The Cancer Genome Atlas.

**FIGURE 4:**
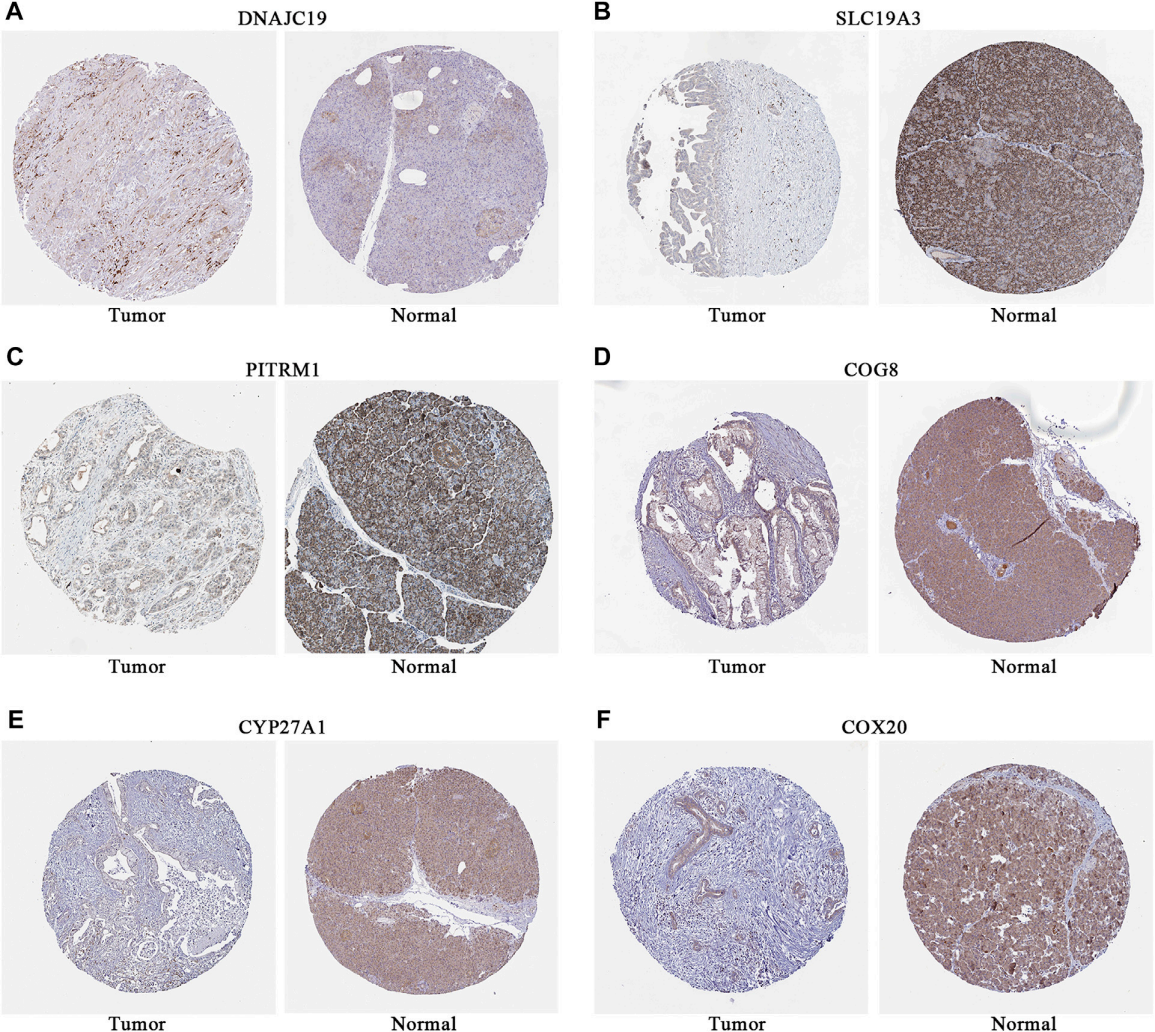
Representative immunostaining images of LMRGs in normal pancreas and PDAC from HPA. **(A)** DNAJC19. **(B)** SLC19A3. **(C)** PITRM1. **(D)** COG8. **(E)** CYP27A1. **(F)** COX20. Brown stained for positive cells; data for NDUFS7 were missing. LMRGs, lactate metabolism-related genes; PDAC, pancreatic ductal adenocarcinoma; HPA, human protein atlas.

To further explore the potential application of the PDAC-specific LMRGs, we evaluated their prognostic potential. Univariate Cox regression analysis revealed that 16 out of the 123 specific LMRGs were significantly associated with prognosis (Figure 2A). After utilizing LASSO regression model to avoid overfitting (Figures 2B, C), a total of 10 LMRGs were subjected to further analysis with a multivariate Cox repression model (Figure 2D) to establish a 7-gene signature that potentially holds specific prognostic values for the management of PDAC patients. Next, we calculated the riskscore of each patient based on the expressional levels of the 7 LMRGs and classified them into two subgroups: low-risk group and high-risk group. Kaplan-Meier survival analysis demonstrated that patients with high riskscore had significantly lower survival probability (Figure 3A). Additionally, PCA and t-SNE analysis indicated that the patients in different risk groups were distributed in two directions (Figures 3B, C). Consistently, patients with high riskscore had a higher probability of death earlier than those with low riskscore, and the 7 LMRGs were differentially expressed in the two groups (Figures 3D–F). Finally, the protein expression levels of the LMRGs in PDAC and normal pancreas were validated in HPA (Figure 4; except for NDUFS7, as data for its expression were missing).

### 3.3 Independent prognostic value of the LMRGs signature

**FIGURE 5:**
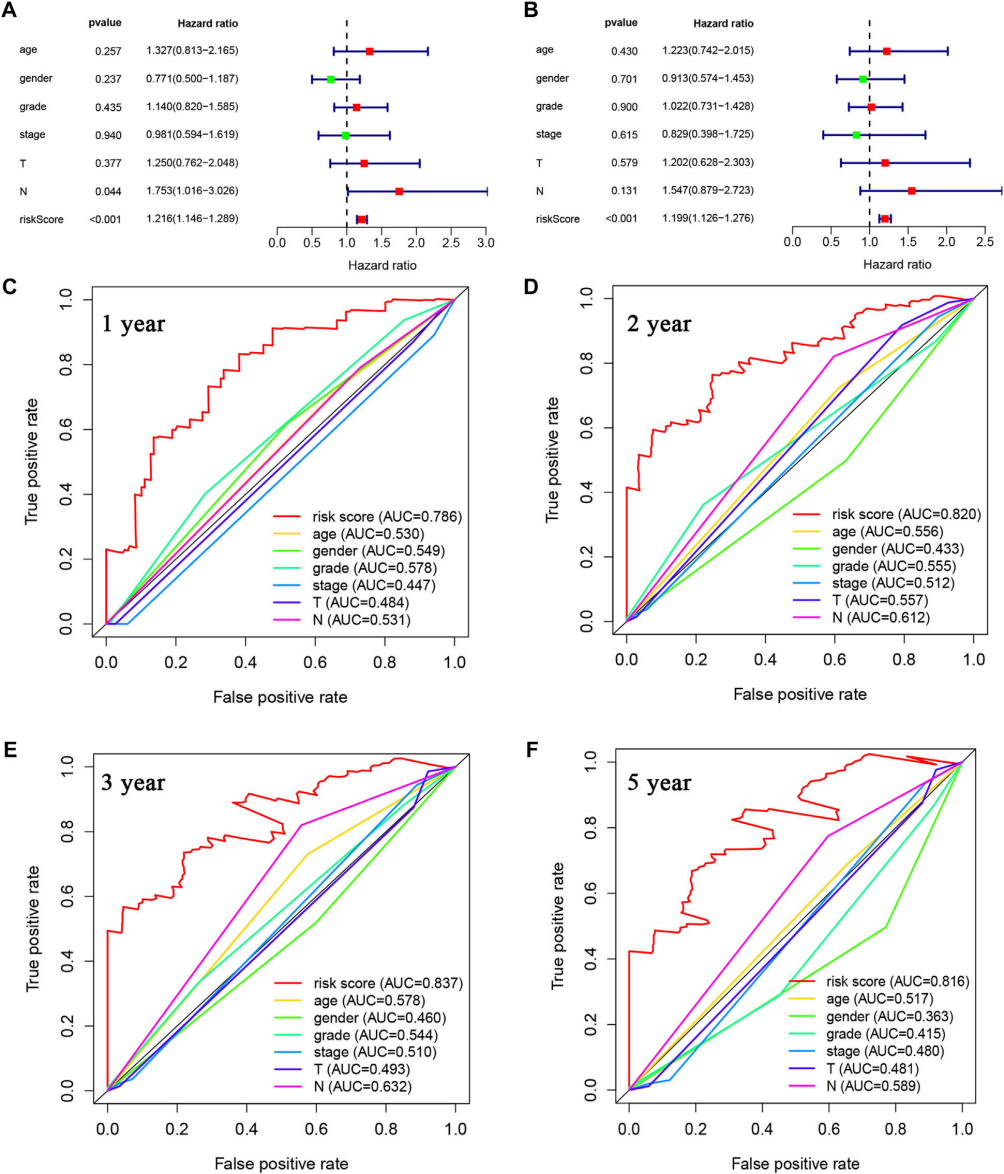
Prognostic value of LMRGs score and other clinical characteristics in PDAC. **(A)** Univariate Cox regression analysis of the riskscore based on the LMRGs signature and other clinical characteristics, including age, gender, grade and stage in PDAC patients. **(B)** Multivariate Cox regression analysis of the riskscore and other clinical characteristics in PDAC patients. **(C–F)** ROC analysis to evaluate the 1-year, 2-year, 3-year, and 5-year OS of PDAC patients. LMRGs, lactate metabolism-related genes; PDAC, pancreatic ductal adenocarcinoma; ROC, receiver operating characteristic; OS, overall survival.

First, univariate and multivariate Cox regression analyses were conducted to determine whether the riskscore was an independent prognostic predictor for OS in PDAC. As depicted in Figures 5A, B, the riskscore demonstrated significant correlation with OS in both univariate and multivariate Cox regression analyses. Moreover, we evaluated the area under the receiver operator characteristic curve (AUC) values of riskscore along with age, gender, grade, stage, T and N status over time periods of 1-, 2-, 3- or 5-year (Figures 5C–F, respectively). Our results indicate that the riskscore calculated from the expressional levels of afore-identified LMRGs might play a vital role in PDAC progression and is an independent predictor of OS.

### 3.4 Establishment of a nomogram for predicting survival in PDAC

**FIGURE 6:**
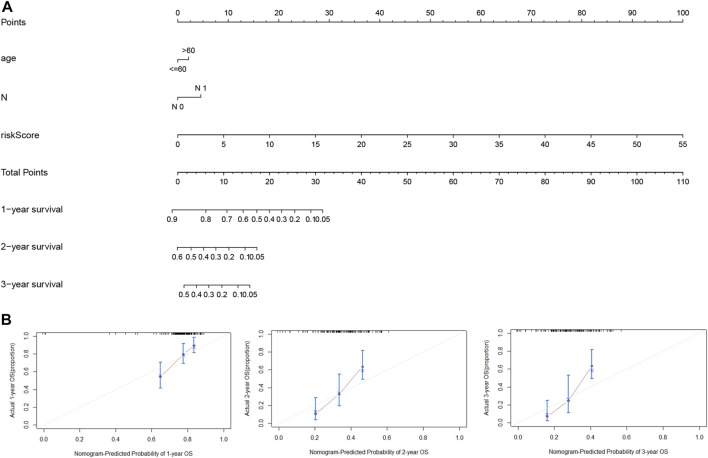
Nomogram for estimation of the survival rate of PDAC patients. **(A)** Development of a nomogram by combining LMRGs score with age and N status to predict the survival probability of PDAC in TCGA dataset. **(B)** Calibration curves of the nomogram for 1-year, 2-year, and 3-year survival. LMRGs, lactate metabolism-related genes; PDAC, pancreatic ductal adenocarcinoma; TCGA, The Cancer Genome Atlas.

For accurate and consistent prediction of overall clinical outcomes, we established a nomogram that takes into consideration of the hazard ratio (HR) of the LMRGs-based riskscore as well as clinical information including age and N (Figure 6A). Not surprisingly, patients older than 60 years or with N1 status had a worse prognosis. Furthermore, the calibration curves in Figure 6B demonstrated a high consistency between the prediction and observation in the TCGA cohort, providing evidence that our model can effectively predict clinical outcomes of PDAC patients.

### 3.5 Predicted functions of LMRGs in PDAC

**FIGURE 7:**
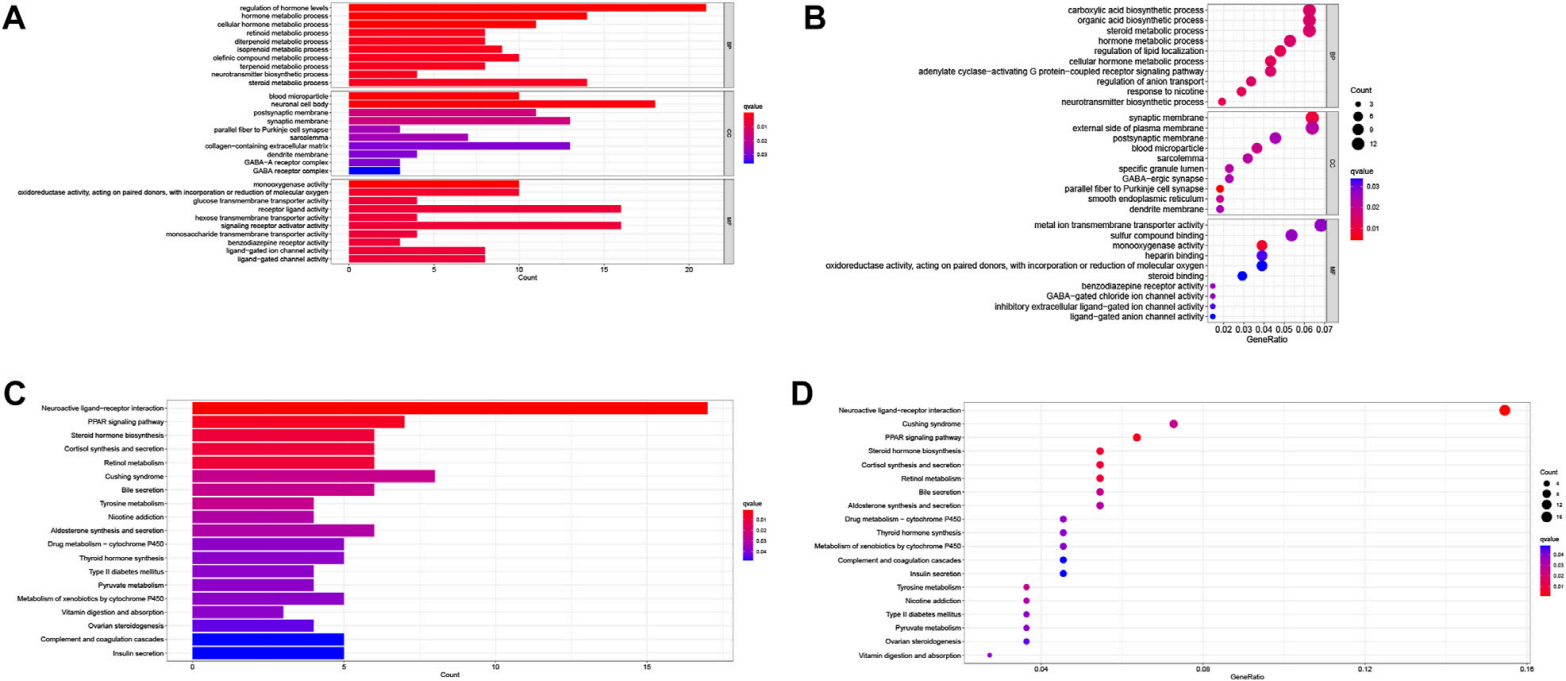
Identification and enrichment analysis of LMRGs in PDAC. **(A,B)** Barplot and bubble map of GO enrichment analysis. **(C,D)** Barplot and bubble map of KEGG pathway enrichment analysis. LMRGs, lactate metabolism-related genes; PDAC, pancreatic ductal adenocarcinoma.

To predict the potential biological functions of these LMRGs, we detected differentially expressed genes between low-risk and high-risk groups using the TCGA cohort. GO pathway analysis (Figures 7A, B) revealed that the LMRGs signature is mainly associated with metabolic and synthetic processes, such as “glucose transmembrane transporter activity” and “hormone metabolic process.” In addition, KEGG pathway analysis (Figures 7C, D) revealed that this LMRGs signature was linked to tumorigenesis signaling cascades, such as “PPAR signaling pathway,” as well as substrate metabolism including pyruvate. Taken together, these results suggested that the PDAC-specific LMRGs signature was associated with tumor metabolism.

### 3.6 Correlation of LMRGs-based signature with sensitivity to gemcitabine in PDAC

**FIGURE 8:**
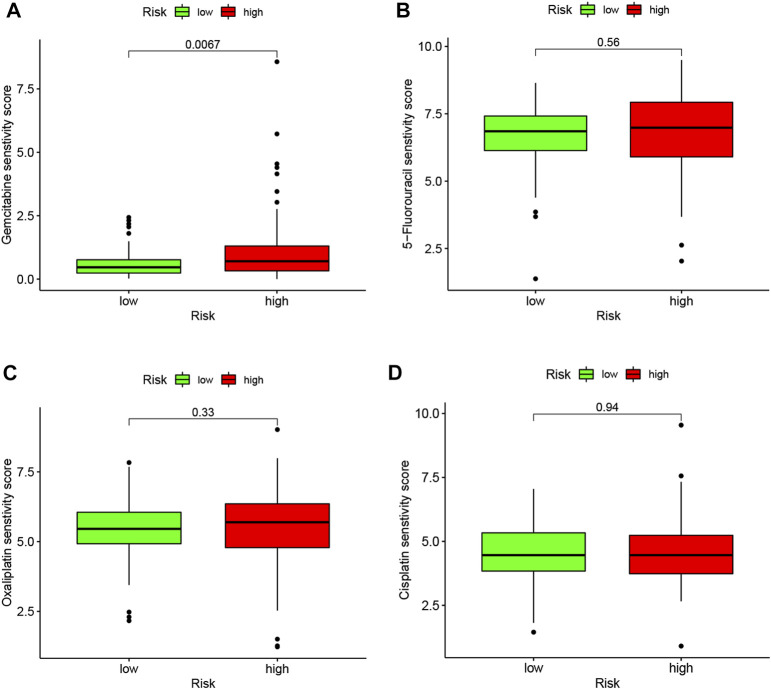
LMRGs signature is associated with chemotherapy response in PDAC. To predict the drug sensitivity of high- and low-risk patients to gemcitabine **(A)**, 5-fluorouracil **(B)**, oxaliplatin **(C)** or cisplatin **(D)**, sensitivity scores were generated by OncoPredict. LMRGs, lactate metabolism-related genes; PDAC, pancreatic ductal adenocarcinoma. Lower score represent higher sensitivity.

To better correlate the LMRGs-based signature with clinical practice, we utilized the “oncoPredict” tool to estimate sensitivity to frequently-used pancreatic cancer chemotherapy agents. Our analysis showed that patients in the low-risk group had higher sensitivity to gemcitabine (Figure 8A). However, there were no significant differences in sensitivity to 5-fluorouracil, oxaliplatin or cisplatin between high-risk and low-risk groups (Figures 8B–D).

### 3.7 Assessment of tumor microenvironment in relation to LMRGs in PDAC

**FIGURE 9:**
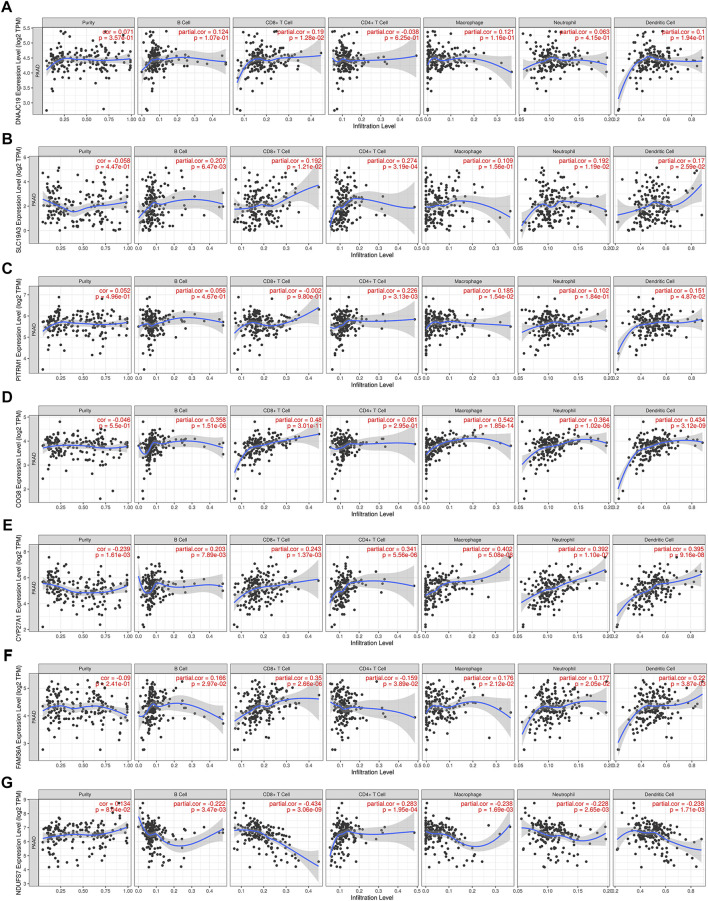
Correlation between expression of LMRGs and tumor purity or immune infiltration levels in PDAC through TIMER. **(A)** Scatter plots showing the correlations between DNAJC19 expression and tumor purity or immune cell infiltration. **(B)** Scatter plots showing the correlations between SLC19A3 expression and tumor purity or immune cell infiltration. **(C)** Scatter plots showing the correlations between PITRM1 expression and tumor purity or immune cell infiltration. **(D)** Scatter plots showing the correlations between COG8 expression and tumor purity or immune cell infiltration. **(E)** Scatter plots showing the correlations between CYP27A1 expression and tumor purity or immune cell infiltration. **(F)** Scatter plots showing the correlations between FAM36A expression and tumor purity or immune cell infiltration. FAM36A is one of aliases for COX20. **(G)** Scatter plots showing the correlations between NDUFS7 expression and tumor purity or immune cell infiltration. LMRGs, lactate metabolism-related genes; PDAC, pancreatic ductal adenocarcinoma; TIMER, Tumor Immune Estimation Resource. *p* < 0.05 was considered statistically significant.

**FIGURE 10:**
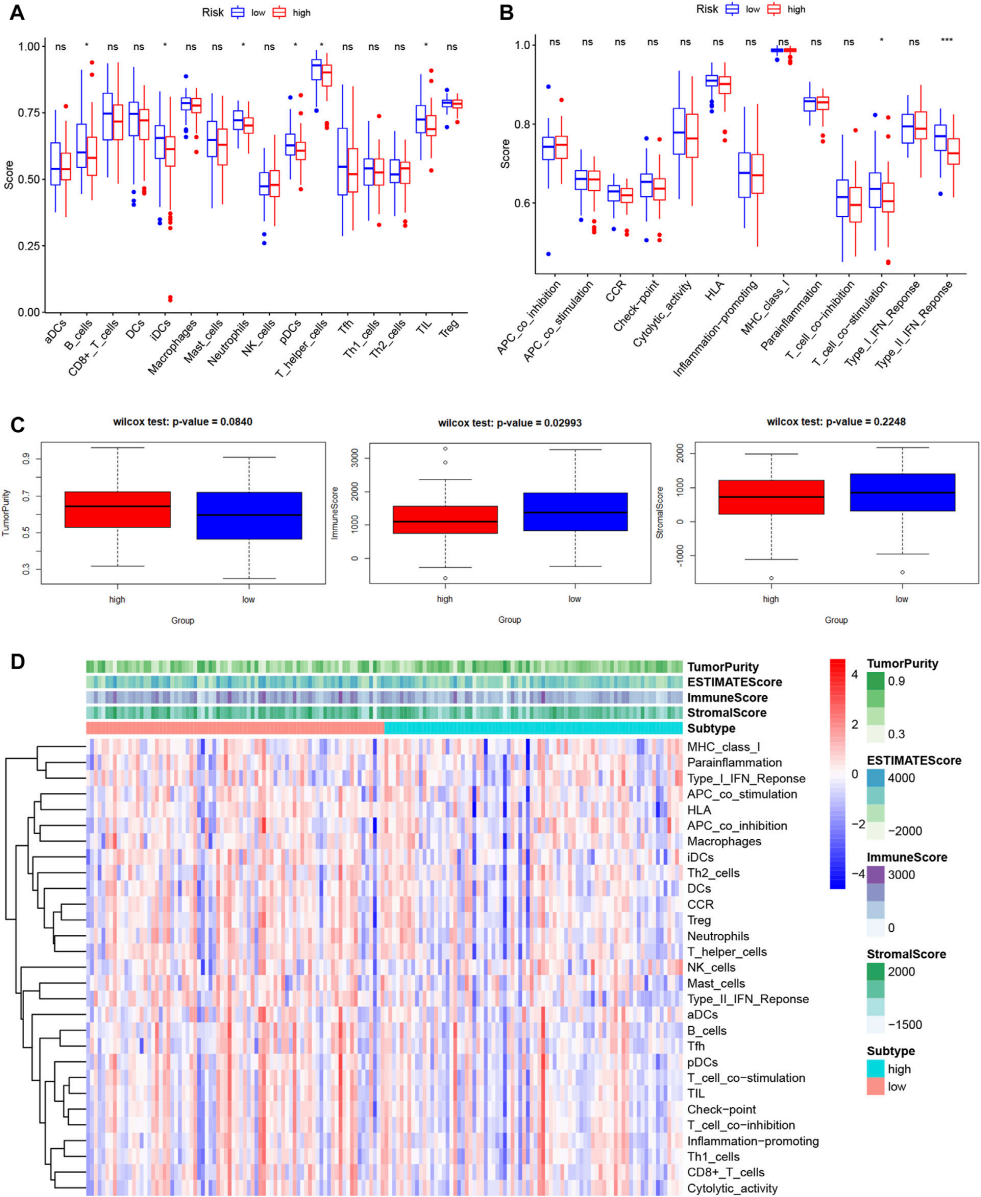
Features of tumor microenvironment in different LMRGs subgroups of PDAC. **(A,B)** Different immune cell infiltration **(A)** or immune cell function **(B)** between low-risk and high-risk groups, which was calculated using ssGSEA algorithm. **(C)** The Boxplots showing the difference in purity, immune score, and stromal score between low-risk and high-risk groups, which was calculated using ESTIMATE algorithm. **(D)** The heatmap displayed the tumor purity, ESTIMATE score, immune score, and stromal score of each PDAC sample. LMRGs, lactate metabolism-related genes; PDAC, pancreatic ductal adenocarcinoma; ssGSEA, single sample gene set enrichment analysis.

Lactate played key roles in cancer progression and immune evasion (de la Cruz-Lopez et al., 2019). Therefore, we explored the correlation between expression of the 7 LMRGs and tumor-infiltrating immune cells in PDAC with TIMER, and found a low correlation in DNAJC19 (Figure 9A; r = 0.19 for CD8^+^ T cells), SLC19A3 (Figure 9B; r = 0.207 for B cell; r = 0.192 for CD8^+^ T cell; r = 0.274 for CD4^+^ T cell; r = 0.192 for neutrophil; r = 0.17 for dendritic cell) and PITRM1 (Figure 9C; r = 0.226 for CD4^+^ T cell; r = 0.185 for macrophage; r = 0.151 for dendritic cell), a medium correlation in COG8 (Figure 9D; r = 0.358 for B cell; r = 0.48 for CD8^+^ T cell; r = 0.542 for macrophage; r = 0.364 for neutrophil; r = 0.434 for dendritic cell), CYP27A1 (Figure 9E; r = 0.203 for B cell; r = 0.243 for CD8^+^ T cell; r = 0.341 for CD4^+^ T cell; r = 0.402 for macrophage; r = 0.392 for neutrophil; r = 0.395 for dendritic cell) and COX20 (Figure 9F; r = 0.166 for B cell; r = 0.35 for CD8^+^ T cell; r = −0.159 for CD4^+^ T cell; r = 0.176 for macrophage; r = 0.177 for neutrophil; r = 0.22 for dendritic cell), whereas a negative correlation in NDUFS7 (Figure 9G; r = −0.222 for B cell; r = −0.434 for CD8^+^ T cell; r = 0.283 for CD4^+^ T cell; r = −0.238 for macrophage; r = −0.228 for neutrophil; r = −0.238 for dendritic cell). Next, we explored the correlation between LMRGs signature and the tumor microenvironment. As for immune cell infiltration, ssGSEA showed that there were more B cells, immature dendritic cells (iDCs), neutrophils, plasmacytoid dendritic cells (pDCs), T helper cells and tumor infiltration lymphocytes (TIL) in the low-risk group (Figure 10A). For immune cell function, “T_cell_co-stimulation” and “Type_II_IFN_Response” were more activated in the low-risk groups (Figure 10B).

In addition, since the tumor microenvironment was dominated by fibroblast in PDAC, we used the “ESTIMATE” algorithm to calculate the stromal and immune scores. Our results indicated that the immune score in low-risk group was significantly higher than that in high-risk group, whereas the between-group difference for the tumor purity or stromal score did not reach significance (Figure 10C). Figure 10D depicted the variation in tumor purity, estimate score, immune score, stromal score, immune cell infiltration and function with increasing riskscore.

### 3.8 Validation of the prognostic value in ICGC datasets

**FIGURE 11:**
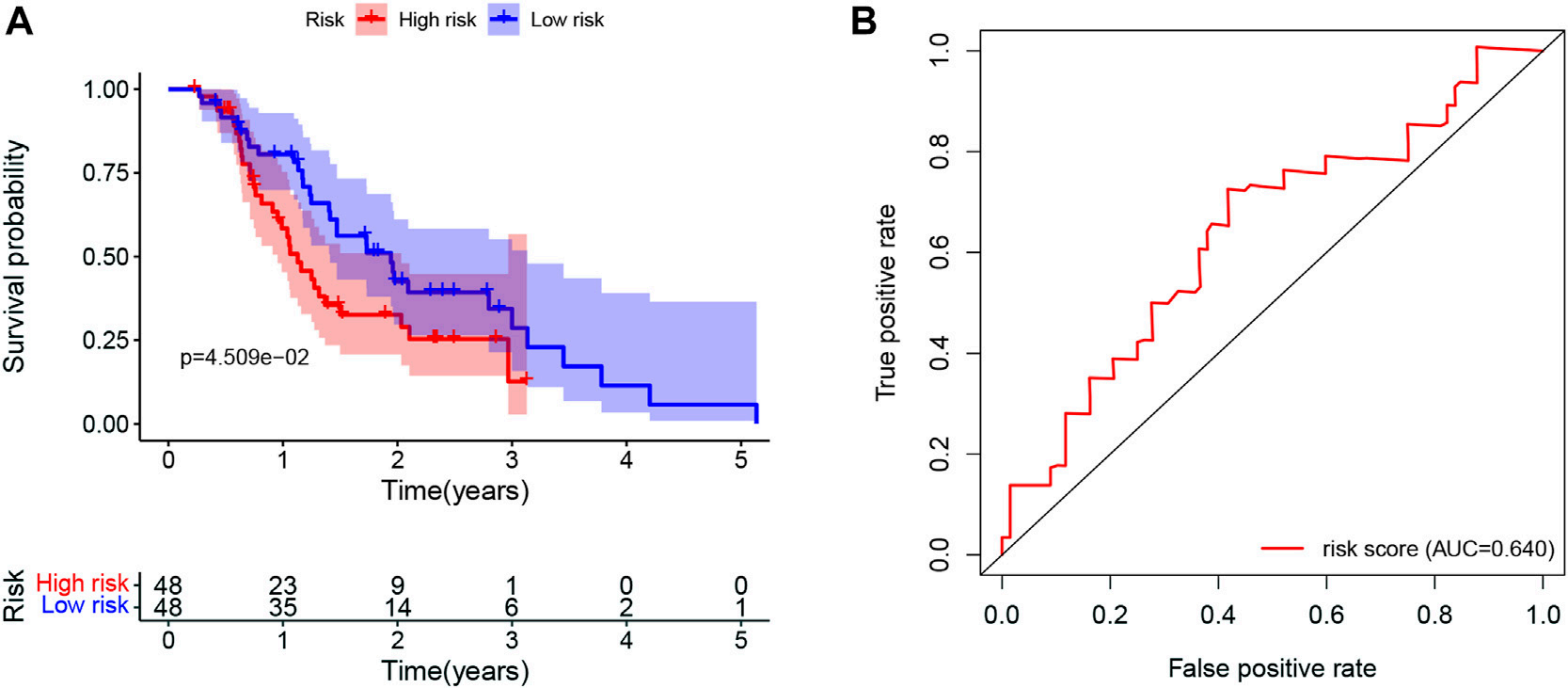
Validation of the prognostic value in ICGC datasets. **(A)** Kaplan-Meier for OS in PDAC patients in ICGC datasets stratified by the mean LMRGs score. **(B)** The ROC curve for 1-year OS in PDAC patients in ICGC datasets, according to the LMRGs signature. ICGC, International Cancer Genome Consortium; OS, overall survival; PDAC, pancreatic ductal adenocarcinoma; LMRGs, lactate metabolism-related genes; ROC, receiver operating characteristic.

The prognostic value of the LMRGs signature was externally validated using ICGC datasets. As shown in Figure 11A, patients in the low-risk group had better OS than those in the high-risk group (*p* < 0.05), with the AUC for 1-year OS being 0.64 (Figure 11B).

## 4 Discussion

PDAC is among the most prevalent and aggressive types of cancer worldwide, with a reported 5-year survival rate of 10% or less (Siegel et al., 2020). Due to lack of symptoms as well as early screening strategies, unfortunately vast majority of patients do not receive the diagnosis until the tumor reaches metastatic stages and become ineligible for surgical resection (Lin et al., 2015). Cytotoxic chemotherapies or radiotherapy was relied on for the treatment of advanced PDAC; however, very modest success has been shown with such treatment strategy (Goess and Friess, 2018). On the other hand, over the last decades, extensive efforts have been made to identify novel prognostic and predictive factors in PDAC, including glypican-1 expressing circulating exosome (Moutinho-Ribeiro et al., 2022), micro- and long-non-coding RNAs (Ashrafizadeh et al., 2022; Prinz et al., 2022), circulating tumorous DNA and cells (Chapin et al., 2022), as well as CA19-9 (Zhao et al., 2022). Notably, CA19-9 is the only FDA-approved and clinically used biomarker. Therefore, identification of novel prognostic and targetable biological markers are warranted to improve the management for PDAC patients.

**FIGURE 12:**
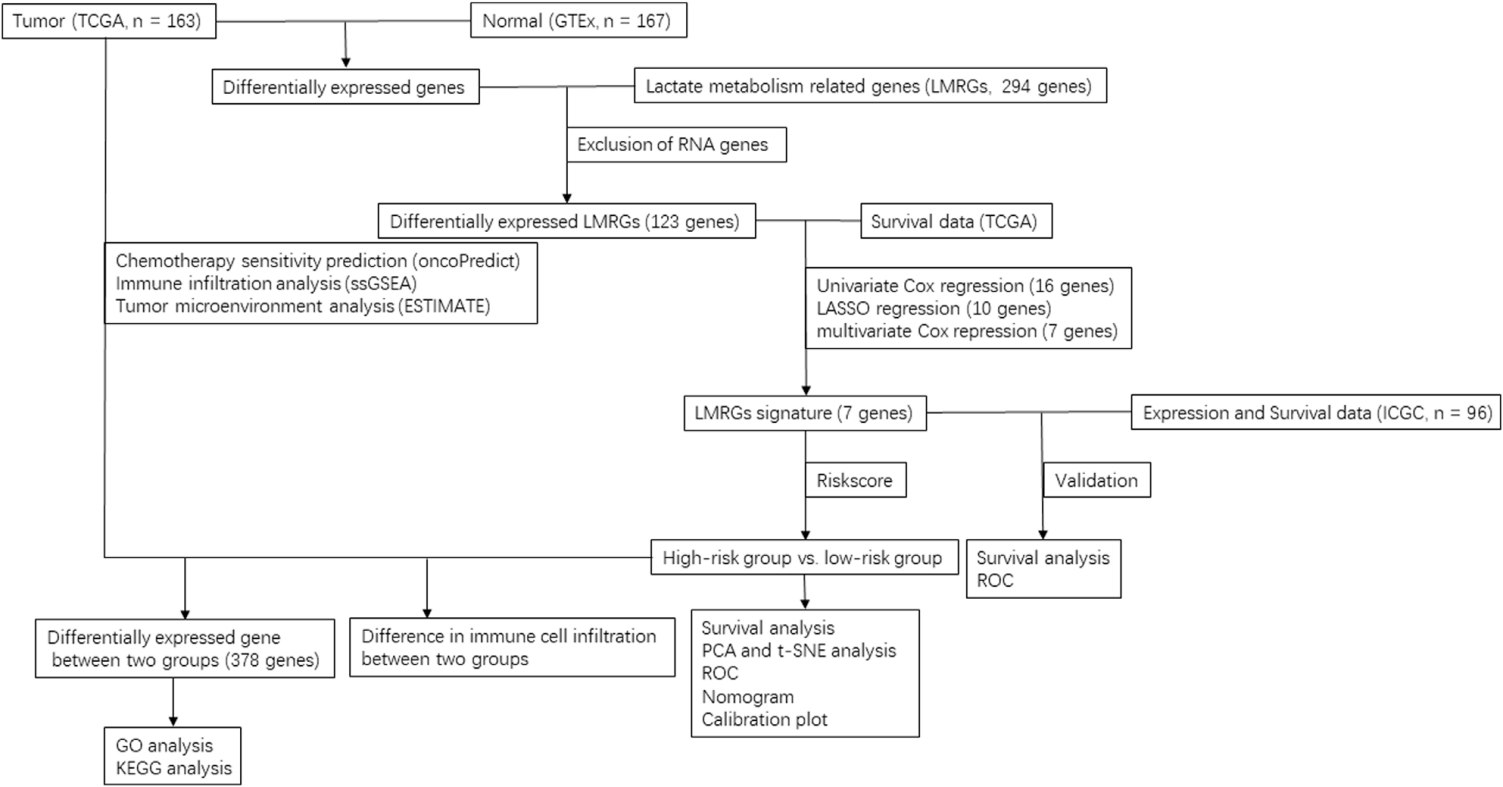
Flow chart of data selection and analysis in this study.

There is a growing body of evidence indicating that lactate homeostasis plays a vital role in the progression of nearly all types of cancers. Cancer cells often rely on aerobic glycolysis to maintain malignant proliferation and growth, which leads to excessive production of lactate, (Hanahan and Weinberg, 2000; Hanahan and Weinberg, 2011). It has been proposed that LMRGs may be involved in the oncogenesis and progression of various types of cancers. Indeed, a LMRGs signature consisted of 6 genes was reported to be independent prognostic predictor for hepatocellular carcinoma (Li et al., 2021). Building on this observation, we aimed to construct a unique LMRGs signature with accurate prognostic value for PDAC patients in the current study (Figure 12).

There have been studies exploring the prognostic value of metabolic-related signature in PDAC (Huo et al., 2021), the authors focused mainly on the metabolic enzymes and transporters, therefore, some LMRGs were not in the list of analyzed genes. In our study, a genetic signature comprised of 7 LMRGs, namely, DNAJC19, SLC19A3, PITRM1, COG8, CYP27A1, COX20 and NDUFS7, was identified through differential analysis (Figure 1) and Cox/LASSO regression analysis (Figure 2). When stratify the current patient population into low- or high-risk subgroups based on the expressional levels of the aforementioned LMRGs, we observed a significantly lower OS in the high-risk patient subgroup, suggesting the prognostic value of the LMRGs signature (Figure 3), in addition, the protein expression of these LMRGs was validated in HPA (Figure 4). More importantly, such prognostic value of the riskscore is independent of other clinical factors including age, gender, grade and tumor stage (Figures 5, 6).

Interestingly, SLC19A3(36) and CYP27A1 (Arem et al., 2015) have been previously implicated in the oncogenesis and progression of PDAC, which partially confirmed the results of our study. Whereas the precise function of DNAJC19, PITRM1, COG8, COX20 or NDUFS7 in PDAC remains unknown. DNAJC19 was the only gene which showed negative correlation with OS (HR 18.059). It plays an important role in mitochondrial protein import machinery in the inner mitochondrial membrane and has been reported to play an important role in cardiomyopathy (Al Tuwaijri et al., 2022; Wachoski-Dark et al., 2022); in non-small-cell lung cancer, DNAJC19 was demonstrated to promote tumor cell growth and metastasis by regulating PI3K/AKT signaling (Zhou et al., 2021). PITRM1 belongs to metallopeptidase and has been reported to be associated with hypoxia and glucose deprivation in glioma cells (Minchenko et al., 2020). COG8 played key roles in protein glycosylation and correlated with favorable OS in kidney renal clear cell carcinoma (Zhang et al., 2021).

Not unexpectedly, genes correlated with the LMRGs signature were enriched in metabolic pathway, such as “pyruvate metabolism” (Figure 7). Since acid tumor microenvironment contributed to chemotherapy and immunotherapy resistance (Wang et al., 2020), we then investigated whether the LMRGs signature is capable of prognosticating the patients’ response to the most commonly-used chemotherapeutic agents in PDAC. Our findings revealed that patients with higher riskscore were less sensitive to gemcitabine, but not 5-fluorouracil, oxaliplatin, or cisplatin (Figure 8), these results suggest that the LMRGs signature may serve as a potential predictive marker for treatment response to gemcitabine in PDAC patients. Moreover, we found that high LMRGs score correlated with low infiltration of effector immune cells and low immune cell function (Figures 9, 10), which was in accordance with other studies that showed lactate plays a key role in constructing the immunosuppressive microenvironment of PDAC (Husain et al., 2013; de la Cruz-Lopez et al., 2019), and it has been previously indicated that SLC19A3 or COG8 was highly associated with immune infiltration in PDAC or dermatomyositis (Huang et al., 2022; Meng et al., 2022)

Finally, the prognostic value of LMRGs signature was validated by using ICGC dataset (Figure 11). Taken together, lower sensitivity to gemcitabine and less immune cell infiltration could partially explain the poor prognosis in the high-risk group.

Collectively, the results of our study indicate that a genomic signature comprised of these LMRGs may be a novel predictor of overall clinical outcomes and holds therapeutic potential for PDAC patients. However, since our data mainly relied on bioinformatics analysis, future experimental studies are warranted to investigate the potential biological mechanisms of these LMRGs, such as DNAJC19, in PDAC.

## Data Availability

Publicly available datasets were analyzed in this study. This data can be found here: TCGA database (https://portal.gdc.cancer.gov/); GTEx database (https://gtexportal.org/); ICGC database (https://dcc.icgc.org/).

## Abbreviations

AUC, area under the receiver operator characteristic curve; CA19-9, carbohydrate antigen 19–9; iDCs, immature dendritic cells; FPKM, fragments per kilobase of exon per million fragments mapped; FDR, false discovery rate; GTEx, genotype-tissue expression; HR, hazard ratio; ICGC, international cancer genome consortium; LMRGs, lactate metabolism-related genes; OS, overall survival; pDCs, plasmacytoid dendritic cells; PDAC, pancreatic ductal adenocarcinoma; ROC, receiver operating characteristic; ssGSEA, single sample gene set enrichment analysis; TIL, tumor infiltration lymphocytes; TCGA, the cancer genome atlas.
