# Supplementary material for: Establishment and validation of a prognostic signature for pancreatic ductal adenocarcinoma based on lactate metabolism-related genes
Source: Front Mol Biosci. 2023 Jun 9;10:1143073. doi: 10.3389/fmolb.2023.1143073 (PMC10288859; doi:10.3389/fmolb.2023.1143073)
Supplement: Supplementary file 1 [file Table1.DOCX]

Table S1 294 lactate metabolism-related genes

ACTN3

HAGH

HIF1A

LDHA

LDHAL6B

LDHB

LDHC

LDHD

MIR210

PARK7

PER2

PFKFB2

PNKD

SLC25A12

TIGAR

TP53

EMB

MYC

SLC16A1

SLC16A3

SLC16A7

SLC16A8

SLC5A12

SLC5A8

LDHAL6A

COG8

COX16

CYP27A1

DNM1L

HPDL

HSD17B10

KARS1

MT-CO1

MT-CO2

MT-CO3

MT-ND1

MT-ND4

MT-ND5

MT-ND6

MT-TF

MT-TH

MT-TL1

MT-TQ

MT-TS1

MT-TS2

MT-TW

MTRFR

NDUFAF3

NDUFB8

NDUFS2

SCO2

SDHB

SQOR

SUCLG1

SURF1

ACAD9

ACADM

B3GALNT2

B4GAT1

CALR

CD46

CFH

CFI

CHEK2

COL4A1

CPT2

CRPPA

DAG1

FKRP

FKTN

FLI1

GAA

GATA1

HBB

HELLPAR

HLA-DRB1

INPP5K

IRAK1

JAK2

KCNN4

KY

LARGE1

LIPA

LYST

MPL

MVK

OCRL

PIEZO1

PIGA

PITRM1

PLA2G6

PLEC

PNPLA2

POMGNT1

POMGNT2

POMK

POMT1

POMT2

RB1

RHAG

RHCE

RHD

RPS14

RXYLT1

SIL1

SLC19A1

SLC25A13

SLC4A1

SLC7A7

SPP1

STAT4

TCIRG1

TET2

USB1

VPS13A

GFM1

MRPL3

NDUFB10

NDUFS4

AIFM1

ALDH4A1

COA8

COQ8A

COX10

COX14

COX15

COX20

COX4I1

COX6B1

COX8A

DARS2

ECHS1

FOXRED1

GFM2

HTRA2

LIPT1

LONP1

LRPPRC

MDH2

MECP2

MPV17

MRPS34

MT-ATP6

MT-ND2

MT-ND3

MT-TK

MT-TN

MT-TV

MTFMT

NARS2

NAXE

NDUFA1

NDUFA10

NDUFA11

NDUFA12

NDUFA13

NDUFA2

NDUFA4

NDUFA6

NDUFA8

NDUFA9

NDUFAF1

NDUFAF2

NDUFAF4

NDUFAF5

NDUFAF6

NDUFAF8

NDUFB11

NDUFB3

NDUFB9

NDUFS1

NDUFS3

NDUFS6

NDUFS7

NDUFS8

NDUFV1

NDUFV2

NUBPL

PDHA1

PDHX

PET100

PET117

PNPT1

RARS2

RMND1

SDHA

SLC13A3

SLC19A3

SLC25A19

SLC39A8

TACO1

TIMMDC1

TMEM126B

TRAPPC12

TRMT10C

TXN2

AARS2

ACAT1

ACAT2

ADAMTS13

AGK

ATAD3A

ATPAF2

BCS1L

C1QBP

CA5A

CARS2

CHCHD10

CLPB

COQ2

COQ4

COQ9

COX5A

COX6A2

CYC1

DGUOK

DLD

DNAJC19

EARS2

FARS2

FASTKD2

FBXL4

FDX2

GOT2

GTPBP3

GYS2

HIBCH

HMGCL

HS6ST2

ISCA1

ISCU

LIAS

LIPT2

LYRM7

MICOS13

MIPEP

MPC1

MRPL12

MRPL44

MRPS14

MRPS16

MRPS22

MRPS28

MT-TI

MT-TL2

MT-TP

MTO1

NDUFC2

NFS1

NGLY1

OGDH

PC

PDP1

PDSS1

PDSS2

PHKG2

PMPCB

PNPLA8

PNPO

POLG

POLG2

PUS1

PYGL

RARS1

RNASEH1

RRM2B

SCO1

SERAC1

SFXN4

SLC25A10

SLC25A26

SLC25A3

SLC25A4

SLC25A42

SOD1

SYNJ1

TANGO2

TARS2

TIMM22

TIMM50

TK2

TMEM70

TRMT5

TRMU

TSFM

TUFM

TWNK

UQCC3

UQCRB

UQCRC2

UQCRQ

WARS2

YARS1

YARS2
